# SAND, a New Protein Family: From Nucleic Acid to Protein Structure and Function Prediction

**DOI:** 10.1002/cfg.93

**Published:** 2001-08

**Authors:** Amanda Cottage, Yvonne J. K. Edwards, Greg Elgar

**Affiliations:** UK Human Genome mapping Project Resource Centre Wellcome Trust Genome Campus Hinxton, Cambridge CB10 1SB UK

## Abstract

As a result of genome, EST and cDNA sequencing projects, there are huge numbers of predicted and/or partially characterised protein sequences compared with a relatively small number of proteins with experimentally determined function and structure. Thus, there is a considerable attention focused on the accurate prediction of gene function and structure from sequence by using bioinformatics. In the course of our analysis of genomic sequence from *Fugu rubripes*, we identified a novel gene, *SAND*, with significant sequence identity to hypothetical proteins predicted in *Saccharomyces cerevisiae, Schizosaccharomyces pombe, Caenorhabditis elegans*, a *Drosophila melanogaster* gene, and mouse and human cDNAs. Here we identify a further *SAND* homologue in human and *Arabidopsis thaliana* by use of standard computational tools. We describe the genomic organisation of *SAND* in these evolutionarily divergent species and identify sequence homologues from EST database searches confirming the expression of SAND in over 20 different eukaryotes. We confirm the expression of two different SAND paralogues in mammals and determine expression of one SAND in other vertebrates and eukaryotes. Furthermore, we predict structural properties of SAND, and characterise conserved sequence motifs in this protein family.


**Comparative and Functional Genomics**
*Comp Funct Genom* 2001; **2**: 226–235.
**DOI:** 10.1002/cfg.93

**Research Article**

### SAND, a new protein family: from nucleic acid to protein structure and function prediction

Amanda Cottage, Yvonne J. K. Edwards and Greg Elgar*
*UK Human Genome mapping Project Resource Centre, Wellcome Trust Genome Campus, Hinxton, Cambridge, CB10 1SB, UK*

*Correspondence to: G. Elgar, UK Human Genome mapping Project Resource Centre, Wellcome Trust Genome Campus, Hinxton, Cambridge, CB10 1SB, UK. E-mail: gelgar@hgmp.mrc.ac.uk*

Received: 1 May 2001
Accepted: 21 June 2001

### Abstract

**As a result of genome, EST and cDNA sequencing projects, there are huge numbers of predicted and/or partially characterised protein sequences compared with a relatively small number of proteins with experimentally determined function and structure. Thus, there is a considerable attention focused on the accurate prediction of gene function and structure from sequence by using bioinformatics. In the course of our analysis of genomic sequence from *Fugu rubripes*, we identified a novel gene, *SAND*, with significant sequence identity to hypothetical proteins predicted in *Saccharomyces cerevisiae*, *Schizosaccharomyces pombe*, *Caenorhabditis elegans*, a *Drosophila melanogaster* gene, and mouse and human cDNAs. Here we identify a further *SAND* homologue in human and *Arabidopsis thaliana* by use of standard computational tools. We describe the genomic organisation of *SAND* in these evolutionarily divergent species and identify sequence homologues from EST database searches confirming the expression of SAND in over 20 different eukaryotes. We confirm the expression of two different SAND paralogues in mammals and determine expression of one SAND in other vertebrates and eukaryotes. Furthermore, we predict structural properties of SAND, and characterise conserved sequence motifs in this protein family. Copyright © 2001 John Wiley & Sons, Ltd.**

**Keywords:** **bioinformatics; structure prediction; comparative sequence analysis; data mining**

### Introduction

Many prokaryotic genomes have now been fully sequenced, as well as several eukaryotic genomes including: *Saccharomyces cerevisiae* (Goffeau *et al.*, 1996; *The yeast genome directory*, 1997), *Caenorhabditis elegans* (The *C.elegans* sequencing consortium, 1998), *Drosophila melanogaster* (Adams *et al.*, 1999) and that of the first plant genome *Arabidopsis thaliana* (Lin *et al.*, 1999; Mayer *et al.*, 1999; Salanoubat *et al.*, 2000; Tabata *et al.*, 2000; Theologis *et al.*, 2000). The first complete vertebrate genomic sequence, that of man, is available but at this time is not fully assembled (Venter *et al* 2001; International human genome sequencing consortium, 2001). Many gaps in the sequence remain to be filled and only two chromosomes are in a finished state (Dunham *et al.*, 1999; Hattori *et al.*, 2000). It is now becoming apparent that the annotation of these genomic sequences relies heavily on gene prediction programs, of which the best only fare reasonably. Continuous sequence similarity searches of large genomic sequences against an increasing pool of cDNAs and ESTs is proving to be costly. A feature of the analysis of these genomes is the number of unknown proteins that they are predicted to encode. For example, in the relatively small genome of *C. elegans*, 60% of predicted genes encoded proteins of unknown function (The *C. elegans* sequencing consortium, 1998). In this study, we have identified a novel gene in the model vertebrate *Fugu rubripes*, which we have called *SAND*, as its location is next to the plasminogen related growth factor receptor (PRGFR) thought to be the orthologue of SEA (EMBL:AJ010317) (Cottage *et al.*, 1999). BLAST (Altschul *et al.*, 1997)

Copyright © 2001 John Wiley & Sons, Ltd.

searches of cDNA and EST sequences have revealed that the *SAND* gene product is expressed in diverse eukaryotes, in various developmental stages in plants, and in many tissue types in vertebrates. We have identified two SAND paralogues in mammals, which appear to be the result of a taxonomy class specific duplication. In an attempt to assign function to this new protein family, we have carried out a computational analysis including similarity searches, multiple sequence alignments, domain identification and characterisation, secondary structure predictions, solvent accessibility predictions and automatic protein fold recognition analyses.

### Materials and methods

### Characterisation of the *F. rubripes* cosmid 165K09

The *F. rubripes* cosmid 165K09 (EMBL : AJ010317) had previously been identified as encoding a PRGFR. The cosmid was sequenced and annotated as reported (Cottage *et al.*, 1999). Additionally, RT PCR was used to verify the predicted *SAND* gene product.

### Bioinformatic analysis of SAND nucleic acid sequences

BLASTn (version 2.0.12) and tBLASTx (version 2.0.12) (Altschul *et al.*, 1997) searches of EMBL release 66 (EMBL) (Stoesser *et al.*, 2001) (and unfinished human genome contigs (**http://www.ncbi.nlm.nih.gov/BLAST/**) and BLASTx (version 2.0.12) (Altschul *et al.*, 1997) searches of SWISS-PROT release 39 (SP) and SWISS-PROT TrEMBL release 16 (SPTR) (Bairoch and Apweiler, 2000) with *F. rubripes SAND* were used to identify homologous sequences. Where gene sequences had not been identified, these were determined from genomic sequences using NIX, a WWW tool to view the results of many DNA analysis programs (Williams, G., Woollard, P. and Hingamp P. unpublished data, **http://www.hgmp.mrc.ac.uk/Registered/Webapp/nix/**). Analysis of the promoter regions, in order to determine potential TATA boxes and conserved transcription factor binding sites, was facilitated by the use of Theatre. Theatre is a tool for obtaining the results for a set of DNA sequences, from various DNA sequence analysis programs (with emphasis of finding transcription factor binding sites), and producing a clear graphical display of this information in a comparative context (Edwards and coworkers; **http://www.hgmp.mrc.ac.uk/Registered/Webapp/theatre/**). tBLASTn version 2.0.12 (Altschul *et al.*, 1997) searches of EST databases were used to generate an *in silico* expression profile of SAND homologues.

### Bioinformatic analysis of SAND protein sequences

When gene sequences had been determined, the derived protein sequences were aligned, together with the mRNA and EST translations using ClustalW version 1.74 (Thompson *et al.*, 1994). Percentage sequence identity and specific conserved residues were determined by the use of Belvu, an alignment viewer written by Erik Sonnhammer (**http://www.cgr.ki.se/cgr/groups/sonnhammer/Belvu.html**). Composite analysis of each protein was performed using PIX, a WWW tool to view the results of many protein analysis programs for a query sequence (**http://www.hgmp.mrc.ac.uk/Registered/Webapp/pix/**). Further database searches using PSI BLAST version 2 (Altschul *et al.*, 1997) were used to identify potential domains by accepting the default and running several iterations. SAND was also submitted for PSI BLAST analysis in overlapping contigs of 100 residues. A consensus secondary structure and associated solvent accessibility was generated using Jpred2 (Cuff *et al.*, 1998) from a ClustalW (Thompson *et al.*, 1994) alignment of seven full length SAND sequences in MSF format. The protein fold recognition program Threader (Jones *et al.*, 1992) was used to score protein sequence compatibility against known protein folds. Sequence threading against a structural databank of 1902 known protein folds was performed for the seven SAND sequences. Threadings were computed in terms of 1. pairwise interaction energies, 2. solvation potential energies and 3. their weighted sum, in order to evaluate the fit of each sand sequence to a particular fold conformation, and represented as Z-scores (=(Energy − Mean)/Standard Deviation). Provided there is greater than 50% sequence and structure matching, the Z scores were sorted for input into a program called SumThreader (Edwards and Perkins, 1996) in order to summarise the outcome of the searches. For each of the three Z scores, the average rank of each fold was calculated from the seven values determined for the individual SAND sequence

Copyright © 2001 John Wiley & Sons, Ltd. 

threadings. The average ranked position for each fold for seven sequences were calculated.

### Results and discussion

### Identifying sequence homologues

The Japanese puffer fish, *F. rubripes*, was chosen as a suitable vertebrate model in which to identify genes of interest. Evidence suggests that the *F. rubripes* gene complement is about the same as that of mammals, and that gene order and structure may be preserved, but within a remarkably compact genome of only 400 Mbp (Brenner *et al.*, 1993). Several gene families have been studied in this vertebrate, including the *Hox* genes (Aparicio *et al.*, 1997), the pheromone receptors (Naito *et al.*, 1998) and the surfeit genes (Armes *et al.*, 1997). Analysis of the genomic sequence from the *F. rubripes* cosmid 165K09 (EMBL : AJ010317) identified a novel sequence, *SAND* (Fr_SAND) (SPTR : Q9YGN1), with sequence identity to hypothetical proteins predicted in *S. cerevisiae* (Sc_SAND) (SP : P53129) and *Sz. pombe* (Sp_SAND) (SP : Q10150), and to part of a hypothetical protein predicted in *C. elegans* (Ce_SAND) (SPTR : Q20298). The *F. rubripes* sequence also has sequence identity to the *D. melanogaster* CG11926 gene product (Dm_SAND) (SPTR : Q9VR38) and a mouse cDNA clone (Mm_SAND1) (EMBL : AK013387). tBLASTx searches of *A. thaliana's* chromosome 2 sequences identified a sequence with similarity to SAND (At_SAND) (EMBL : AC006283). Two human SAND homologues were identified: a cDNA clone KIAA0872 (Hs_SAND2) (SPTR : O94949), and a predicted open reading frame on chromosome 3p21 (Hs_SAND1) (EMBL: AC068701). Whilst BLAST (Altschul *et al.*, 1997) searches reveal the expression of at least two SAND genes in a number of mammals (see Table 1A and 1B), including human, mouse, pig and cow, only one SAND gene can be detected in any non-mammalian organism (see Table 1C).

### Intron/exon structure

*SAND1* is predicted to comprise two exons in *Sz. pombe* (see Table 2A) and one in *S. cerevisiae*, however the hypothetical protein produced from the reported *S. cerevisiae* prediction introduces gaps into an alignment with other SAND proteins. The *S. cerevisiae* sequence is approximately 644 residues by comparison to the 520 residues encoded by the other sequences. A better alignment with the other SAND sequences was achieved by removing 54 residues from the N terminus and introducing an intron which removes residues 537–577 (SP : P53129), experimental confirmation is needed of this prediction and this sequence was not used for secondary structure predictions.

Gene sequences were determined from the *C. elegans* genomic data by Genefinder (P. Green and L. Hillier unpublished software). These predictions were confirmed or adjusted to account for protein, cDNA and EST matches (The *C. elegans* sequencing consortium, 1998). The *C. elegans* SAND

Table 1. Expression profile of SAND generated by EST database searches

1A. Expression profile of SAND1 in mammals

| Organism | EMBL Accession number | Expression |
|---|---|---|
| *Homo sapiens* | CNSLT1EA9 | Placenta |
| | CNSLT1FVF | T cells/T cell leukemia |
| | BE793894 | Lung |
| *Mus musculus* | BF321829 | Mammary infiltrating ductal carcinoma |
| | BF179283 | Mammary, gross tissue |
| | BF584835 | Colon |
| | AI853696 | Brain |
| | AU06752 | Brain |
| *Rattus norvegicus* | AI237869 | Normalized spleen |
| | BE111711 | Cardiac tissue |
| *Sus scrofa* | BE234656 | Pooled |
| | BF193484 | Pooled |
| *Bos taurus* | AW655266 | Pooled |
| | AW653632 | Pooled |
| | BF664794 | Pooled |

1B. Expression profile of SAND2 in mammals

| Organism | EMBL Accession number | Expression |
|---|---|---|
| *Homo sapiens* | BE785702 | Lung |
| | BF313087 | Neuroblastoma |
| | BE885699 | Leiomyosarcoma |
| | CNSLTOYVF | T cell leukemia |
| | AK023374 | Ovarian tumour |
| *Mus musculus* | AW626447 | Lung |
| | AW322902 | Lung |
| *Sus scrofa* | AW785756 | Pooled |
| | AW322902 | Pooled |
| *Bos taurus* | BF602206 | Pooled |

Copyright © 2001 John Wiley & Sons, Ltd. 

1C. Expression profile of SAND in non mammalian eukaryotes

| Organism | EMBL accession number | Expression |
|---|---|---|
| *Gallus gallus* | GGA395913 | Bursa of fabricus |
| | GGA392697 | Bursa of fabricus |
| *Danio rerio* | AW343114 | Mixed tissue |
| | AW115566 | Mixed tissue |
| *Drosophila melanogaster* | AI294091 | Larvae |
| | AI516700 | Embryo 0–24 hrs |
| | AI389145 | Head |
| *Caenorhabditis elegans* | CE12C12 | Mixed |
| | AU111676 | Whole |
| | AU112292 | Whole |
| *Dictyostelium discoidium* | DDC4407 | Gamete |
| | DDC4408 | Gamete |
| *Arabidopsis thaliana* | BE522449 | Developing seeds |
| | AV536086 | Flower buds |
| | AV555362 | Green siliques |
| *Glycine max* | AW201401 | Cotyledons |
| *Lycopersicon esculentum* | AI777002 | Callus |
| | AW222181 | Pericarp |
| | AW222182 | Fruit |
| | AI487936 | Ovary |
| *Medicago truncatula* | CN506BLP | Arbuscular mycorrhiza |
| *Solanum Tuberosum* | BF459706 | Tuber |
| *Pinus taeda* | BG318737 | Xylem |
| *Secale cereale* | BE588169 | Root tip |
| *Gossypium hirsutum* | AWI87646 | Cotton fibre |
| *Hordeum vulgare* | BG300416 | Seedling shoot |

protein (F41H10.4 SP:Q20298) is predicted by Genefinder to be encoded by 13 exons; however it would appear that exons 1–4 belong to a neighbouring gene and it is this sequence that shows similarity to a *Plasmodium yoelii* rhoptry protein as specified in the annotation. Residues encoded by exon 8 introduced a gap in an alignment with other SAND proteins, so this was removed from the gene prediction. We therefore expected that *C. elegans* SAND is encoded by 6 exons, although the exact gene start could not be determined from the given sequence, possibly due to sequencing/assembly errors and so is not shown in Figure 1.

The predicted gene structure of *D. melanogaster* did not require further adjustment. The *D. melanogaster* genome annotation used Genscan (Burge and Karlin, 1997), and a version of Genie that uses expressed sequence tag data (Reese *et al.*, 2000), plus the results of cDNA and protein database searches, followed by review by human annotators (Adams *et al.*, 2000). *D. melanogaster* SAND is encoded by three exons. In contrast, SAND is encoded by 12 exons in *A. thaliana*; the genes small intron and exon sizes, coupled with low G+C content (42%), may explain why this gene was missed by the gene prediction programs used in this genomic project, even though the annotation involved both DNA and protein database searches and gene prediction with the programs Genscan (Burge and Karlin, 1997), Genefinder (P. Green and Hillier unpublished) and Grail (Uberbacher *et al.*, 1991).

Human and mouse SANDs have the highest G+C content (>60%), followed by *F. rubripes* (53%) and *D. melanogaster*. *C. elegans*, both yeast and *A. thaliana* all have a lower G+C composite at

Table 2. Comparison of intron/exon sizes and exon phase between *SAND* sequences in *A. thaliana* (At), *C. elegans* (Ce), *F. rubripes* (Fr), Human (Hs), *D. melanogaster* (Dm), *S. cerevisiae* (Sc) and *S. pombe* (Sp). The question mark indicates unresolved or ambiguous boundaries in gene annotation

2A. Size of exons in base pairs

| Ex. | At | Ce | Fr | Hs1 | Hs2 | Dm | Sc | Sp |
|---|---|---|---|---|---|---|---|---|
| 1 | 186 | 155? | 124 | 127 | 148 | 593 | 1449? | 1295 |
| 2 | 101 | 213 | 384 | 486 | 327 | 152 | 204 | 247 |
| 3 | 106? | 155 | 766 | 766 | 820 | 842 | | |
| 4 | 72 | 371 | 148 | 148 | 148 | | | |
| 5 | 166 | 407 | 143 | 141 | 201 | | | |
| 6 | 122 | 277 | | | | | | |
| 7 | 233 | | | | | | | |
| 8 | 83 | | | | | | | |
| 9 | 263 | | | | | | | |
| 10 | 92 | | | | | | | |
| 11 | 65 | | | | | | | |
| 12 | 96 | | | | | | | |

2B. Size of introns in base pairs

| Ex. | At | Ce | Fr | Hs1 | Hs2 | Dm | Sc | Sp |
|---|---|---|---|---|---|---|---|---|
| A | 57 | 269? | 146 | 1208 | 1818 | 66 | 120 | 86 |
| B | 66 | 58 | 89 | 637 | 557 | 56 | | |
| C | 122 | 298 | 129 | 718 | 380 | | | |
| D | 249 | 50 | 439 | 98 | 2425 | | | |
| E | 96 | 51 | | | | | | |
| F | 366 | | | | | | | |
| G | 162 | | | | | | | |
| H | 251 | | | | | | | |
| I | 81 | | | | | | | |
| J | 90 | | | | | | | |
| K | 103 | | | | | | | |

Copyright © 2001 John Wiley & Sons, Ltd. 

2C. Phase of introns

| At | Ce | Fr | Hs1 | Hs2 | Dm | Sc | Sp |
|---|---|---|---|---|---|---|---|
| 0 | II | I | I | I | II | 0 | II |
| II | II | I | I | I | I | | |
| II | I | II | II | II | | | |
| II | 0 | 0 | 0 | 0 | | | |
| 0 | II | | | | | | |
| II | | | | | | | |
| I | | | | | | | |
| 0 | | | | | | | |
| II | | | | | | | |
| I | | | | | | | |
| 0 | | | | | | | |

around 40%. Analysis of the promoter regions by the use of Theatre (Edwards and coworkers; unpublished material) determined that the promoters appear to be largely TATA-less; no significant sequence identity could be determined between them. Comparisons between the promoter regions proved difficult due to the variable length of the N terminus of the encoded proteins and the lack of sequence identity between them across this region.

Both intron/exon number and size, as well as intron phase, show no conservation between yeasts, fly, worm and plant (see Table 2). Analyses of *SAND* in these organisms may elucidate some interesting clues to the dynamics of intron formation/loss. Vertebrate evolution, however, appears to have taken a more concerted route: *F. rubripes* SAND and human SAND1 and SAND2 are all encoded in five exons, and the intron phases of the three genes are conserved (see Table 2C). Exon 4 is the same size in all three genes Fr_SAND (148 bp), Hs_SAND1 (148 bp) and Hs_SAND2 (148 bp) (see Table 2A).

### Genomic loci

The orientation of genes neighbouring SAND and their accession numbers are given in Table 3. Human SAND2 protein KIAA0872 has been mapped to chromosome 16 (Nagase *et al.*, 1998). BLAST (Altschul *et al.*, 1997) searches of the human SAND2 locus (EMBL:AC009139) have shown its 5′ neighbour on the direct strand to be a putative pheromone receptor, whilst its 3′ neighbour on the reverse strand encodes a hypothetical protein. Hypothetical proteins are also predicted as neighbours to SAND in *D. melanogaster*, *C. elegans*, and *A. thaliana*, but these sequences do not appear to be homologous. Of interest are the ribosomal proteins predicted 5′ to SAND and on the opposite strand in both *D. melanogaster* and *S. cerevisiae*. The 5′ neighbour (on the reverse strand) of *F. rubripes SAND* is the TRAF interacting protein TRIP. The 3′ neighbour, on the direct strand, is the plasminogen related growth factor receptor (PRGFR3). This gene organisation is conserved on the genomic segment of chromosome 3p21 encoding human SAND1. Human TRIP (SPTR:O00467) is on the reverse strand 5′ of SAND1, and the plasminogen related growth factor receptor (SP:Q04912) is approximately 10Kbp on the direct strand 3′ to it. Considerable interest has been focused on 3p21 in man as a cancer hot spot; it is particularly implicated in all major types of lung cancer (Kok *et al.*, 1987).

### Expression profile

Expression of the human SAND protein KIAA0872 has been assayed by means of RT PCR from mRNA derived from heart, brain, lung, liver, smooth muscle, kidney, pancreas, spleen, testis and ovary. KIAA0872 was shown to be expressed predominately in brain, kidney and ovary (Nagase *et al.*, 1998). In this study, RT PCR analysis confirmed expression of *F. rubripes* SAND in brain, kidney and ovary, reflecting the expression profile seen in human. Although some expression was seen in other tissues assayed such as heart, lung, liver, smooth muscle, spleen and testis. An expression profile was generated for the SAND transcripts by BLAST (Altschul *et al.*, 1997) searches with all identified SAND sequences and is shown in Tables 1A, 1B and 1C. Human SAND2 (KIA00872) had been identified in pooled tissues as well as lung. The transcript had also been identified in four neoplastic tissues, of particular interest is the lack of exon 3 from EMBL:AK023374 identified from an ovarian tumour. Expression of Hs_SAND1 appears more widespread and includes neurological, lymphatic and cardiac tissue, whilst once again neoplastic tissues are also represented. The expression of SAND in other eukaryotes appears to occur at various developmental stages, and even in specialised tissues such as the bursa of fabricus in chicken. In plants, expression of SAND is seen in both monocots and dicots, and is even to be found expressed in the xylem of pine. SAND is expressed in many different tissue types in plants, including: flowers, seeds, tubers, leaves, shoots and roots.

Copyright © 2001 John Wiley & Sons, Ltd.

Copyright © 2001 John Wiley & Sons, Ltd.

Table 3. Characterisation of SAND loci

| | 5′ | | 3′ | |
|---|---|---|---|---|
| | **Accession number** | **Identification** | **Accession number** | **Identification** |
| Fr_SAND | SPTR:Q9YGN2 | TRIP (−) | SPTR:Q9YGN0 | PRGFR3 (+) |
| Dm_SAND | SPTR:Q9VR37 | Ribosomal protein S2 (−) | SPTR:Q9VR39 | Hypothetical protein (+) |
| Ce_SAND | SPTR:Q20297 | Hypothetical protein (+) | SPTR:Q20299 | Similarity to GRM5 (+) |
| Sc_SAND | SP:P25443 | Ribosomal protein S4 (−) | SP:P53128 | Met1 (−) |
| Sp_SAND | SPTR:Q10147 | Probable T complex protein (+) | EMBL:Z69239.1 | p65 (+) |
| At_SAND | SPTR:AAD20686 | Hypothetical protein (+) | SPTR:AAD20658 | Hypothetical protein (+) |
| Hs_SAND2 | EM: AC009139 | Probable pheromone receptor (+) | EM: AC009139 | Hypothetical protein(−) |
| Hs_SAND1 | SPTR:O00467 | TRIP (−) | SP:Q04912 | MSP Receptor RON (+) |

The orientation of the gene neighbouring SAND is given in parenthesis, (+) for the leading strand and (–) for the reverse strand.

### Protein structure and function

Inferring function from sequence data is the new dogma in DNA research, and the main focus of molecular biologists and bioinformaticians following in the wake of the large genomic projects. Despite current innovations in bioinformatics, this endeavour is non trivial and requires validation.

### Search for a structural homologue using basic search methods

In order to determine if SAND has sequence similarity to proteins with known 3D structures, BLASTP (version 2.0.12) (Altschul *et al.*, 1997) was used to search NRL-3D (**http://pir.georgetown.edu/pirwww/dbinfo/nrl3d.html**), a databank of protein sequences whose structures have been experimentally determined (Garavelli *et al.*, 2001). No structural homologues were identified by searches with the seven full SAND sequences reported in this communication.

PSI-BLAST (Altschul *et al.*, 1997) – Position Specific Iterated BLAST uses an iterative search in which sequences identified in the first round of searching are used to build a score model for the next round. PSI BLAST has been used to successfully identify homologous protein sequences that have known 3D structures, even when the query and subject sequences have less than 20% sequence identity overall (Bork *et al.*, 1999). Successive PSI BLAST 2 iterations with the seven full length SAND protein sequences resulted in convergence after the 4th iteration.

### Search against the databank of Pfam profiles

Profile Hidden Markov Models (HMMs) built from Pfam alignments can be useful for automatically recognising that a new protein contains an existing protein domain, even if the sequence similarity is weak. Pfam HMMs (Sonnhammer *et al.*, 1998) were searched with the nine SAND sequences using the search package HMMER; no significant matches were reported. A seed alignment of SAND is available in PfamB (pfam b8448) but only contains 5 partial sequences from *H. sapiens* (SAND2), *F. rubripes*, *C. elegans*, *Sz. pombe* and *S. cerevisiae*. No homologous structural domains are reported for these proteins.

### PIX analysis of the nine SAND protein sequences

Composite analysis of the SAND proteins for protein motifs and structural elements was facilitated by using PIX (**http://www.hgmp.mrc.ac.uk/Registered/Webapp/pix/**). Matches to various

**Figure 1.** A ClustalW alignment of seven full length SAND amino acid sequences. The following abbreviations are used Hs (*H. sapiens*), Fr (*F. rubripes*), Dm (*D. melanogaster*), At (*A. thaliana*) Mm (*Mus musculus*) and Sp (*S. pombe*). Shown below the alignment is the Jpred2 consensus secondary structure prediction (H – helix, E – strand, – loop) and solvent accessibility (b – buried, e – exposed). Shown in bold underlined type are two cysteines located on helix A10 and strand B12, they are present only in vertebrates and yeast, they may form a disulphide bridge in the mature peptide. SAND has 13 conserved sequence motifs. Motif 3 contains 4 and 6 hydrophobic residues which coincide with beta strands B4 and B5. Motif 5 contains the conserved residues NYDLRRL encoded on loop L9 which are also present in 1pii. Motif 13 contains charged C terminal residues predicted on loop L26

Copyright © 2001 John Wiley & Sons, Ltd. 

features, for example transmembrane domains, coiled coils, signal peptides and peptide cleavage sites were reported in individual sequences, but the threshold at which these features were determined was not significant. Furthermore, these features were not present in the majority of the sequences. With the exception of *A. thaliana*, PSORT kNN predictions only marginally predict SAND as a nuclear localised protein ahead of the prediction as a cytoplasmic protein. The *A. thaliana* SAND is predicted as a plasma membrane located protein with a score of 42%. There were no significant matches to domain databases, e.g. SBASE (Murvai *et al.*, 2001), ProDom (Corpet *et al.*, 1998), BLOCKS (Henikoff *et al.*, 1999), PRINTS (Attwood *et al.*, 1997) and PROSITE (Hofmann *et al.*, 1999). Although comprehensive protein database searches have not revealed a possible function for the SAND proteins, they firmly establish SAND as a new protein family.

### Alignment and prediction of secondary structure and solvent accessibility

The *C. elegans* and *S. cerevisiae* SAND sequences were not included in the alignment (Figure 1) due to problems with their annotations as outlined in this manuscript. The alignment comprises seven sequences that have low sequence identity overall, this typically being less than 30% between pairs of sequences. There is no shared sequence similarity at their N terminus (sequence lengths vary between 104–151 residues). However, following the N terminus, several regions of highly conserved residues are apparent (see Figure 1). These provide evidence for at least two domains within the SAND proteins.

Jpred2 was used to predict the secondary structure and solvent accessibility of the SAND proteins. Three types of input can be supplied to Jpred; a single protein sequence, an unaligned set of protein sequences, or a multiple protein sequence aligned in MSF format. The ClustalW alignment shown in Figure 1 was used as input to Jpred. The Jpred2 consensus secondary structure and solvent accessibility predictions are also shown in Figure 1. The N terminal region described previously is predicted not to contain either alpha helices or beta strands. The SAND proteins are predicted to contain thirteen alpha helices and thirteen beta strands, plus a total of 27 loops. All beta strands are predicted to be largely solvent inaccessible, as well as four helices A2, A7, A8 and A9. Eight of the thirteen helices display an amphipathic pattern (A3, A4, A5, A6, A10, A11, A12 and A13); these helices are likely to be located on the outer surface of the protein with one side of the helix facing the solvent and the other the hydrophobic interior. The lengths of secondary structure elements predicted are typical of those observed in known protein structures and A3 is the only helix predicted to be over 20 residues. Given that the proteins share a low level of sequence identity, the accuracy of the prediction will be affected. The Jpred secondary structure prediction is expected to be about 70% accurate at the amino-acid residue level (Cuff *et al.*, 1998).

### Automatic protein fold recognition

The identification of a protein fold was attempted using Threader (Jones *et al.*, 1992). SumThreader (Edwards and Perkins, 1996) was used to summarise the outputs. The sequences in Figure 1 minus the N terminal residues with the absence of regular secondary structure predicted were used as input for threading analysis.

The SAND sequences were matched 'favourably' with protein structures (protein structure codes and domain annotations are given and the average Z score are given in parentheses): 1pii00(2.65), 1pdz02(2.65), 1eedP0(2.62), 2exo00 (2.56), 1mpp00 (2.55) and 1hpm00(2.52). The Z scores of these matches approach the threshold score of 2.7. In the Threader user guide, this score is specified as "borderline significant, possibly correct". 1pii consists of two alpha beta TIM barrel domains. 1pii is the experimentally determined structure of the bifunctional *E. coli* enzyme phosphoribosylanthranilate isomerase: indoleglycerolphosphate synthase. The 1pdz lyase and the 2exo hydrolase structures both adopt an alpha beta TIM barrel fold. 1hpm is a heat shock protein ATPase fragment adopting an open alpha beta alpha sandwich structure. 1eedP0 and 1mpp00 are aspartate proteinases, adopting beta barrel structures. This beta barrel structure is not compatible with the SAND secondary structure prediction results, that comprise equal numbers of alpha helices and beta strands. The predicted secondary structure of SAND is only a fair match with the largely alternating pattern of helices and strands of 1pii. However, the secondary structure and solvent accessibility predictions for SAND indicate mostly amphipathic helices and predominately solvent

Copyright © 2001 John Wiley & Sons, Ltd.

inaccessible strands, these characteristics are typical properties observed in known alpha beta protein fold types like the alpha beta sandwich structures or the closed alpha beta TIM barrels.

### Conclusions

Lack of conformity and standards in annotation has resulted in variability between genomes in how accurately genes are predicted. In this example, SAND has been partially missed from the *A. thaliana* genome annotation, mis-predicted in *C. elegans* and *S. cerevisiae*, whilst Hs_SAND1 has been not annotated in the human genome. Whilst there are at least two mammalian SAND genes, and at least one SAND gene is present in all eukaryotes, the function of these genes' products is still completely unknown. It is possible that at least one of the mammalian paralogues may play a role in neoplastic disease following loss of exon 3. Using bioinformatic analyses, this study has illustrated the complexities of determining what the structure and function of these proteins might be. When faced with a totally new gene coding for a protein product of unknown function, this task is non trivial and in this example after considerable database searching and analysis we have made only small advances in this endeavour. SAND is only one gene product in approximately 60% of the 19, 000 in *C. elegans* that have an unknown function. A complete eukaryotic genome sequence is today's achievement, but a functional understanding of it is still a distant goal.

### References

No authors listed. 1997. The yeast genome directory. *Nature* **387**(suppl.): 5.

Adams MD, Celniker SE, Holt RA, *et al.* 2000. The genome sequence of Drosophila melanogaster. *Science* **287**: 2185–2195.

Altschul SF, Madden TL, Schaffer AA, *et al.* 1997. Gapped BLAST and PSI-BLAST: a new generation of protein database search programs. *Nucleic Acids Res* **25**: 3389–3402.

Aparicio S, Hawker K, Cottage A, *et al.* 1997. Organization of the Fugu rubripes Hox clusters: evidence for continuing evolution of vertebrate Hox complexes. *Nat Genet* **16**: 79–83.

Armes N, Gilley J, Fried M. 1997. The comparative genomic structure and sequence of the surfeit gene homologs in the puffer fish Fugu rubripes and their association with CpG-rich islands. *Genome Res* **7**: 1138–1152.

Attwood TK, Avison H, Beck ME, *et al.* 1997. The PRINTS database of protein fingerprints: A novel information resource for computational molecular biology. *J Chem Inf Comput Sci* **37**: 417–424.

Bairoch A, Apweiler R. 2000. The SWISS-PROT protein sequence database and its supplement TrEMBL. *Nucleic Acids Res* **28**: 45–48.

Bork P, Doerks T, Springer TA, Snel B. 1999. Domains in plexins: links to integrins and transcription factors. *Trends Biochem Sci* **24**: 261–263.

Brenner S, Elgar G, Sandford R, Macrae A, Venkatesh B, Aparicio S. 1993. Characterization of the pufferfish Fugu genome as a compact model vertebrate genome. *Nature* **366**: 265–268.

Burge C, Karlin S. 1997. Prediction of complete gene structures in human genomic DNA. *J Mol Biol* **268**: 78–94.

Corpet F, Gouzy J, Kahn D. 1998. The ProDom database of protein domain families. *Nucleic Acids Res* **26**: 323–326.

Cottage A, Clark M, Hawker K, *et al.* 1999. Three receptor genes for plasminogen related growth factors in the genome of the puffer fish *Fugu rubripes*. *FEBS Lett* **443**: 370–374.

Cuff JA, Clamp ME, Siddiqui AS, Finlay M, Barton GJ. 1998. JPred: a consensus secondary structure prediction server. *Bioinformatics* **14**: 892–893.

Dunham I, Shimizu N, Roe BA, *et al.* 1999. The DNA sequence of human chromosome 22. *Nature* **402**: 489–495.

Edwards YJK, Perkins SJ. 1996. Assessment of protein fold predictions from sequence information: the predicted alpha/beta doubly wound fold of the von Willebrand factor type A domain is similar to its crystal structure. *J Mol Biol* **2602**: 277–285.

Garavelli JS, Hou ZL, Pattabiraman N, Stephens RM. 2001. The RESID Database of protein structure modifications and the NRL-3D Sequence-Structure Database. *Nucleic Acids Res* **29**: 199–201.

Goffeau A, Barrell BG, Bussey H, *et al.* 1996. Life with 6000 genes. *Science* **274**: 563–567.

Hattori M, Fujiyama A, Taylor TD, *et al.* 2000. The DNA sequence of human chromosome 21. The chromosome 21 mapping and sequencing consortium. *Nature* **405**: 311–319.

Henikoff S, Henikoff JG, Pietrokovski S. 1999. Blocks+: a non-redundant database of protein alignment blocks derived from multiple compilations. *Bioinformatics* **15**: 471–479.

Hofmann K, Bucher P, Falquet L, Bairoch A. 1999. The PROSITE database, its status in 1999. *Nucleic Acids Res* **27**: 215–219.

Jones DT, Taylor WR, Thornton JM. 1992. A new approach to protein fold recognition. *Nature* **358**: 86–89.

Kok K, Osinga J, Carritt B, *et al.* 1987. Deletion of a DNA sequence at the chromosomal region 3p21 in all major types of lung cancer. *Nature* **330**: 578–581.

Lin X, Kaul S, Rounsley S, *et al.* 1999. Sequence and analysis of chromosome 2 of the plant Arabidopsis thaliana. *Nature* **402**: 761–768.

Mayer K, Schuller C, Wambutt R, *et al.* 1999. Sequence and analysis of chromosome 4 of the plant Arabidopsis thaliana. *Nature* **402**: 769–777.

Murvai J, Vlahovicek K, Barta E, Pongor S. 2001. The SBASE protein domain library, release 8.0: a collection of annotated protein sequence segments. *Nucleic Acids Res* **29**: 58–60.

Nagase T, Ishikawa K, Suyama M, *et al.* 1998. Prediction of the coding sequences of unidentified human genes. XII. The complete sequences of 100 new cDNA clones from brain which code for large proteins in vitro. *DNA Res* **5**: 355–364.

Naito T, Saito Y, Yamamoto J, *et al.* 1998. Putative pheromone

Copyright © 2001 John Wiley & Sons, Ltd. 

receptors related to the Ca2+-sensing receptor in Fugu. *Proc Natl Acad Sci U S A* **95**: 5178–5181.

Reese MG, Kulp D, Tammana H, Haussler D. 2000. Genie--gene finding in Drosophila melanogaster. *Genome Res* **10**: 529–538.

Salanoubat M, Lemcke K, Rieger M, *et al.* 2000. Sequence and analysis of chromosome 3 of the plant Arabidopsis thaliana. *Nature* **408**: 820–822.

Sonnhammer EL, Eddy SR, Birney E, Bateman A, Durbin R. 1998. Pfam: multiple sequence alignments and HMM-profiles of protein domains. *Nucleic Acids Res* **261**: 320–322.

Stoesser G, Baker W, van den Broek A, *et al.* 2001. The EMBL Nucleotide Sequence Database. *Nucleic Acids Res* **29**: 17–21.

Tabata S, Kaneko T, Nakamura Y, *et al.* 2000. Sequence and analysis of chromosome 5 of the plant Arabidopsis thaliana. *Nature* **408**: 823–826.

The C. elegans Sequencing Consortium. 1999. Genome sequence of the nematode C. elegans: a platform for investigating biology. *Science* **282**: 2012–2018.

The International Human Genome Mapping Consortium. 2001. A physical map of the human genome. *Nature* **409**: 934–941.

Theologis A, Ecker JR, Palm CJ, *et al.* 2000. Sequence and analysis of chromosome 1 of the plant Arabidopsis thaliana. *Nature* **408**: 816–820.

Thompson JD, Higgins DG, Gibson TJ. 1994. CLUSTAL W: improving the sensitivity of progressive multiple sequence alignment through sequence weighting position-specific gap penalties and weight matrix choice. *Nucleic Acids Res* **22**: 4673–4680.

Uberbacher EC, Mural RJ. 1991. Locating protein-coding regions in human DNA sequences by a multiple sensor-neural network approach. *Proc Natl Acad Sci U S A* **8824**: 11261–11265.

Venter JC, Adams MD, Myers EW, *et al.* 2001. The sequence of the human genome. *Science* **291**: 1304–1351.

*The*

**Genomics**

*Website at Wiley*

**www.wiley.co.uk/genomics**

The **Genomics** website at Wiley is a new and **DYNAMIC** resource for the genomics community, offering **FREE** special feature articles and new information **EACH MONTH**.

Find out more about *Comparative and Functional Genomics*, and how to view all articles published this year **FREE OF CHARGE**!

Visit the **Library** for hot books in Genomics, Bioinformatics, Molecular Genetics and more.

Click on **Primary Research** for information on all our up-to-the minute journals, including: *Genesis*, *Bioessays*, *Gene Function and Disease*, and the *Journal of Gene Medicine*.

Let the **Genomics** website at Wiley be your **guide** to genomics-related web sites, manufacturers and suppliers, and a calendar of conferences.

WILEY

1516

Copyright © 2001 John Wiley & Sons, Ltd.

## References

[PDF_00683] Adams M. D., Celniker S. E., Holt R. A., Evans C. A., Gocayne J. D., Amanatides P. G., Scherer S. E., Li P. W., Hoskins R. A., Galle R. F. (2000). The genome sequence of Drosophila melanogaster.. Science.

[PDF_00685] Altschul S. F., Madden T. L., Schäffer A. A., Zhang J., Zhang Z., Miller W., Lipman D. J. (1997). Gapped BLAST and PSI-BLAST: a new generation of protein database search programs.. Nucleic Acids Res.

[PDF_00688] Aparicio S., Hawker K., Cottage A., Mikawa Y., Zuo L., Venkatesh B., Chen E., Krumlauf R., Brenner S. (1997). Organization of the Fugu rubripes Hox clusters: evidence for continuing evolution of vertebrate Hox complexes.. Nat Genet.

[PDF_00691] Armes N., Gilley J., Fried M. (1997). The comparative genomic structure and sequence of the surfeit gene homologs in the puffer fish Fugu rubripes and their association with CpG-rich islands.. Genome Res.

[PDF_00695] Attwood T. K., Avison H., Beck M. E., Bewley M., Bleasby A. J., Brewster F., Cooper P., Degtyarenko K., Geddes A. J., Flower D. R. (1997). The PRINTS database of protein fingerprints: a novel information resource for computational molecular biology.. J Chem Inf Comput Sci.

[PDF_00699] Bairoch A., Apweiler R. (2000). The SWISS-PROT protein sequence database and its supplement TrEMBL in 2000.. Nucleic Acids Res.

[PDF_00702] Bork P., Doerks T., Springer T. A., Snel B. (1999). Domains in plexins: links to integrins and transcription factors.. Trends Biochem Sci.

[PDF_00705] Brenner S., Elgar G., Sandford R., Macrae A., Venkatesh B., Aparicio S. (1993). Characterization of the pufferfish (Fugu) genome as a compact model vertebrate genome.. Nature.

[PDF_00709] Burge C., Karlin S. (1997). Prediction of complete gene structures in human genomic DNA.. J Mol Biol.

[PDF_00777] C. elegans Sequencing Consortium (1998). Genome sequence of the nematode C. elegans: a platform for investigating biology.. Science.

[PDF_00711] Corpet F., Gouzy J., Kahn D. (1998). The ProDom database of protein domain families.. Nucleic Acids Res.

[PDF_00713] Cottage A., Clark M., Hawker K., Umrania Y., Wheller D., Bishop M., Elgar G. (1999). Three receptor genes for plasminogen related growth factors in the genome of the puffer fish Fugu rubripes.. FEBS Lett.

[PDF_00716] Cuff J. A., Clamp M. E., Siddiqui A. S., Finlay M., Barton G. J. (1998). JPred: a consensus secondary structure prediction server.. Bioinformatics.

[PDF_00719] Dunham I., Shimizu N., Roe B. A., Chissoe S., Hunt A. R., Collins J. E., Bruskiewich R., Beare D. M., Clamp M., Smink L. J. (1999). The DNA sequence of human chromosome 22.. Nature.

[PDF_00721] Edwards Y. J., Perkins S. J. (1996). Assessment of protein fold predictions from sequence information: the predicted alpha/beta doubly wound fold of the von Willebrand factor type A domain is similar to its crystal structure.. J Mol Biol.

[PDF_00726] Garavelli J. S., Hou Z., Pattabiraman N., Stephens R. M. (2001). The RESID Database of protein structure modifications and the NRL-3D Sequence-Structure Database.. Nucleic Acids Res.

[PDF_00730] Goffeau A., Barrell B. G., Bussey H., Davis R. W., Dujon B., Feldmann H., Galibert F., Hoheisel J. D., Jacq C., Johnston M. (1996). Life with 6000 genes.. Science.

[PDF_00732] Hattori M., Fujiyama A., Taylor T. D., Watanabe H., Yada T., Park H. S., Toyoda A., Ishii K., Totoki Y., Choi D. K. (2000). The DNA sequence of human chromosome 21.. Nature.

[PDF_00735] Henikoff S., Henikoff J. G., Pietrokovski S. (1999). Blocks+: a non-redundant database of protein alignment blocks derived from multiple compilations.. Bioinformatics.

[PDF_00738] Hofmann K., Bucher P., Falquet L., Bairoch A. (1999). The PROSITE database, its status in 1999.. Nucleic Acids Res.

[PDF_00741] Jones D. T., Taylor W. R., Thornton J. M. (1992). A new approach to protein fold recognition.. Nature.

[PDF_00743] Kok K., Osinga J., Carritt B., Davis M. B., van der Hout A. H., van der Veen A. Y., Landsvater R. M., de Leij L. F., Berendsen H. H., Postmus P. E. (1987). Deletion of a DNA sequence at the chromosomal region 3p21 in all major types of lung cancer.. Nature.

[PDF_00746] Lin X., Kaul S., Rounsley S., Shea T. P., Benito M. I., Town C. D., Fujii C. Y., Mason T., Bowman C. L., Barnstead M. (1999). Sequence and analysis of chromosome 2 of the plant Arabidopsis thaliana.. Nature.

[PDF_00749] Mayer K., Schüller C., Wambutt R., Murphy G., Volckaert G., Pohl T., Düsterhöft A., Stiekema W., Entian K. D., Terryn N. (1999). Sequence and analysis of chromosome 4 of the plant Arabidopsis thaliana.. Nature.

[PDF_00780] McPherson J. D., Marra M., Hillier L., Waterston R. H., Chinwalla A., Wallis J., Sekhon M., Wylie K., Mardis E. R., Wilson R. K. (2001). A physical map of the human genome.. Nature.

[PDF_00752] Murvai J., Vlahovicek K., Barta E., Pongor S. (2001). The SBASE protein domain library, release 8.0: a collection of annotated protein sequence segments.. Nucleic Acids Res.

[PDF_00755] Nagase T., Ishikawa K., Suyama M., Kikuno R., Hirosawa M., Miyajima N., Tanaka A., Kotani H., Nomura N., Ohara O. (1998). Prediction of the coding sequences of unidentified human genes. XII. The complete sequences of 100 new cDNA clones from brain which code for large proteins in vitro.. DNA Res.

[PDF_00764] Reese M. G., Kulp D., Tammana H., Haussler D. (2000). Genie--gene finding in Drosophila melanogaster.. Genome Res.

[PDF_00766] Salanoubat M., Lemcke K., Rieger M., Ansorge W., Unseld M., Fartmann B., Valle G., Blöcker H., Perez-Alonso M., Obermaier B. (2000). Sequence and analysis of chromosome 3 of the plant Arabidopsis thaliana.. Nature.

[PDF_00769] Sonnhammer E. L., Eddy S. R., Birney E., Bateman A., Durbin R. (1998). Pfam: multiple sequence alignments and HMM-profiles of protein domains.. Nucleic Acids Res.

[PDF_00772] Stoesser G., Baker W., van den Broek A., Camon E., Garcia-Pastor M., Kanz C., Kulikova T., Lombard V., Lopez R., Parkinson H. (2001). The EMBL nucleotide sequence database.. Nucleic Acids Res.

[PDF_00774] Tabata S., Kaneko T., Nakamura Y., Kotani H., Kato T., Asamizu E., Miyajima N., Sasamoto S., Kimura T., Hosouchi T. (2000). Sequence and analysis of chromosome 5 of the plant Arabidopsis thaliana.. Nature.

[PDF_00782] Theologis A., Ecker J. R., Palm C. J., Federspiel N. A., Kaul S., White O., Alonso J., Altafi H., Araujo R., Bowman C. L. (2000). Sequence and analysis of chromosome 1 of the plant Arabidopsis thaliana.. Nature.

[PDF_00785] Thompson J. D., Higgins D. G., Gibson T. J. (1994). CLUSTAL W: improving the sensitivity of progressive multiple sequence alignment through sequence weighting, position-specific gap penalties and weight matrix choice.. Nucleic Acids Res.

[PDF_00790] Uberbacher E. C., Mural R. J. (1991). Locating protein-coding regions in human DNA sequences by a multiple sensor-neural network approach.. Proc Natl Acad Sci U S A.

[PDF_00794] Venter J. C., Adams M. D., Myers E. W., Li P. W., Mural R. J., Sutton G. G., Smith H. O., Yandell M., Evans C. A., Holt R. A. (2001). The sequence of the human genome.. Science.

